# On the Diversity of Symptoms in Scarlatina Maligna

**Published:** 1857-11

**Authors:** George Sutton

**Affiliations:** Aurora, Dearborn County, Indiana


ART. II.—*Observations on the Diversity of Symptoms in Scarlatina Maligna.* By GEORGE SUTTON, M.D., of Aurora, Dearborn County, Indiana.

THERE are few diseases that Western physicians have to encounter, that are more terribly fatal than the malignant form of scarlatina. Prevailing at different periods, under different grades of violence, we see scarlatina at one time in an epidemic form, so mild as scarcely to require the aid of the physician, and at another, in a character so malignant as to baffle all medical treatment and equal the most deadly epidemics in the rapidity with which it destroys life. Why this disease should, at different periods, assume such different forms in the same localities, apparently under precisely the same circumstances, must, I suppose, be attributed to those unknown causes which favor the spread of epidemics over the globe.

Within the last twenty years scarlatina has prevailed in the southeastern portion of Indiana, at different times, under the most opposite forms of violence. A brief review of the epidemics of this disease, which have prevailed in Dearborn County since 1836, may probably afford some interest, as showing the periods in which scarlatina has assumed, in this section of country, the mild, the inflammatory, and malignant varieties.

Dr. Drake tells us, in the sixth volume of the Western Journal of Medicine, page 348, that scarlatina prevailed extensively throughout the valley of the Ohio, in the year 1831. That year, I have been credibly informed, scarlatina prevailed as an epidemic in Dearborn County, and assumed at Hartford, in this county, its most malignant form. In the fall and winter of 1837, the disease prevailed as an epidemic in the northern portion of the county; it presented an inflammatory form, and appeared to require an antiphlogistic course of treatment. This epidemic did not prevail at Aurora, or in the southern part of the county. In 1845 scarlatina prevailed (although not extensively), in the neighborhoods of Aurora and Wilmington; it assumed the mild and anginose varieties; but few deaths occurred from the disease, and it was seldom difficult to manage. In 1847 notices appeared in the public papers of the prevalence of scarlatina, in a malignant form, in different portions of the State. In the spring of 1848 it was prevailing in Dearborn County, and on the 22d of August it suddenly appeared at Aurora, and prevailed in a form entirely different from that it had ever assumed in this town, or even this section of country before. The first cases were in two families who resided within a few feet of each other, on the side of the public highway, near the outskirts of the town. A child in each family was attacked the same day. The disease at first appeared to spread from these cases as if by infection, but it soon became epidemic, and we were then unable to perceive, in many instances, any connection between the cases that occurred. It continued with great malignancy during the winter, generally appearing with fresh violence just before damp or wet weather, and gradually subsided and disappeared in the latter part of February. Nearly every case was asthenic in its character: in some instances it was rapidly fatal, death occurring in from eight to twelve hours after the attack. It presented a variety of symptoms, sometimes so opposite in their character as almost to appear like different diseases: it was principally confined to children, but no age was exempt. Adults were frequently attacked with scarlatina faucium, and I thought that we had evidence that it was occasionally spread by this form of the disease. It was highly infectious, and there were instances in which the infection appeared to be communicated by the clothing of persons who had been attending on the sick. These were some of the prominent features which this terrible epidemic presented.

Scarlatina prevailed at this time in a malignant form at Lawrenceburg, also in Switzerland County. In a letter which I received from Dr. Wm. Tate, of Lawrenceburg, he writes, "that out of twenty cases of malignant scarlatina, which at that time (Nov. 19th) had come under his notice, fifteen had proved fatal. Of nineteen cases of scarlatina anginosum, four were fatal; of ten of scarlatina simplex, none, of course, were fatal." We had but few cases during this epidemic that were mild. Shortly before the disappearance of scarlatina, measles began to prevail not only in Aurora, but in the surrounding country. The latter assumed an asthenic or malignant form, and continued in force during the months of March and April, and disappeared in May. On the 11th of June, cholera existed at Aurora as an epidemic. It was supposed to have originated from some cases left by steamboats, as they ascended the Ohio River. This disease continued to prevail, in its most deadly form, for six weeks, destroying, in some of the squares, more than half the inhabitants. Why all these diseases should have assumed such malignancy at this period it is difficult to tell. There were no local causes that would be more likely to develop the malignant forms of disease at that period than any other. The condition of our town at that time was similar to what it has since been, when scarlatina assumed its mildest form. In the spring and summer of 1853 scarlatina appeared again at Aurora as an epidemic; also in different parts of the county. At Aurora the disease presented the malignant, the anginose, and mild varieties. Although we had many cases of the malignant form of the disease, still this variety was not so common as it was in 1848–9. In the fall of 1856, after an unusually dry and healthy summer, scarlatina again made its appearance at Aurora: it continued to prevail during the winter and disappeared in the spring. This time the malady assumed an unusually mild form; a large proportion of cases did not require medical treatment. About the middle of March, measles began to prevail; since that time this disease has prevailed more extensively in this section of country than it has done *for the last twenty years*, but in a remarkably mild form. During the summer, pertussis has also prevailed extensively throughout this section of country. The continual influx of strangers into the town during the construction of the Ohio and Mississippi Railroad, may probably have favored the frequent prevalence of scarlatina as an epidemic, as there were but few children in Aurora, after the epidemics of 1848–9, and 1853, that had not been attacked with this disease.

Wilson, in his work on diseases of the skin, states that "It is deserving of remark, that the disease (scarlatina) is not always confined to the human race;" that "during the prevalence of this epidemic it appears that horses suffered from a similar affection, colts from acute enteritis; cattle, sheep, and pigs from phlyctenoid fever, the latter diseases being very probably the epizootic which occasioned so much devastation in England during the past year."

It is worthy of notice, since exanthematous diseases have recently been so prevalent in this section of country, that during last summer and fall, there existed a remarkable epizootic amongst the hogs, not only in Dearborn County but in other portions of the State; also in Ohio and Kentucky. The disease spread over the country, and these animals died by hundreds and thousands. Some of our farmers lost almost their entire stock—from seventy to eighty out of the hundred. This malady had some appearance of an exanthematous disease; but I think that it has not its exact resemblance amongst the diseases to which the human system is subject. From some experiments and examinations that I made, for the purpose of ascertaining whether this epizootic resembled any of our epidemic diseases, I came to the conclusion that the malady was intermediate between erysipelas and some of the specific eruptive diseases, partaking of the nature of each: it was, like the eruptive diseases, highly infectious, the infection having a period of incubation of about thirteen days; and the animal had the disease but once: but, like erysipelas, it presented a form of diffusive inflammation, which spread along the different tissues, presenting a great variety of symptoms; sometimes it attacked the mucous membrane of the alimentary canal, producing diarrhœa, which has caused the disease to receive the name of "Hog Cholera." It is a singular fact, that the healthy summer of 1856 should have given rise to so fatal a disease amongst some of our inferior animals. Whether exanthematous diseases have existed over the whole country, in which this disease amongst the hogs has prevailed, I cannot say.

During the epidemics of scarlatina, in 1848–9 and 1853, I frequently met with the violent and opposite forms which this disease sometimes presents, and which, I think, have not been as fully described in our medical books as they deserve. From notes which I took at the time, the malignant variety of scarlatina may, for the sake of description and directing attention to its different forms, be subdivided into the four following modifications:

1st. Where the system is suddenly prostrated at the commencement of the disease, as if from a severe shock upon the organic nervous system.

2d. Where the violence of the disease is directed to the brain, producing congestion or inflammation of that organ.

3d. Where the alimentary canal is the principal seat of irritation, producing symptoms resembling a violent cholera morbus.

4th. Where the disease is principally directed to the throat and respiratory passages.

These appeared to be the principal modifications of scarlatina maligna, which, either single or under different combinations, were present in every case that I have met with.

A few remarks upon these subdivisions will show, I think, the symptoms in each to be as distinct as those upon which is founded the division into the mild, the anginose, and malignant varieties.

The attack in the first form is generally sudden; sometimes there are, for twenty-four or forty-eight hours previous, the usual premonitory symptoms of scarlatina, but no symptoms calculated to create alarm. The child, probably, although feeling unwell, may be at play or walking about, when it is suddenly seized with sickness at the stomach, and vomits, or feels faint; then the most alarming symptoms soon become manifest and rapidly increase. The pulse soon becomes extremely frequent, small, and easily compressed. The respiration is irregular, the countenance pale and deathlike, and as the disease advances, the eyes become sunken and inexpressive, and the skin cold. The throat is sometimes but slightly inflamed. The tongue is generally moist, and covered at first with a thick coat. Sometimes the patient is restless, at others inclined to doze, but is easily aroused, and answers questions correctly. Different grades of reaction occasionally take place. In some instances the rash, of a purple color, appears and disappears, and the patient lingers for several days. I have seen reaction take place, the rash come out over the body, and the patient recover after there had been the most alarming symptoms. In other instances the disease was very rapid, death occurring in from eight to twelve hours after the attack. In this form of the malady, the throat was frequently but little affected, and there was sometimes but little cerebral disturbance. The whole nervous system seemed to be suddenly depressed, the symptoms resembling those in which the impression is more upon the *heart* than upon the extreme *bloodvessels*, producing effects more nearly allied to syncope than congestion.

In all of our epidemics, we have met with a number of cases where the violence of the disease was directed to the brain. This modification of scarlatina is familiar to every physician. It may be in the form of active congestion, producing convulsions, or in a sthenic or asthenic form of inflammation. Tweedie tells us, in his article on scarlatina, that "When there have been violent delirium and other symptoms of cerebral excitement, the arachnoid membrane is vascular, or even opaque, with effusion of a serous or sometimes milky fluid underneath. In such cases, the substance of the brain is also unusually vascular." The symptoms of inflammation of the brain or its membranes are often prominent, and form a marked contrast with the first modification of the disease. We have, in this cerebral form, fever, pain in the head, continued delirium, throbbing of the carotids, quick pulse, flushed face, suffused eyes, intolerance of light, &c.; almost every symptom different from those presented in the other form. The fauces in this variety are not always much inflamed. The skin is dry, and patches of rash may be seen over different parts of the body, sometimes florid, at others of a dark color. Convulsions or coma generally terminate the case. Convulsions occurring in the early stages of the disease, depending upon active congestion, may be followed by inflammation of the brain, and the symptoms mentioned. In some instances the convulsions are followed by coma, small and irregular pulse, livid appearance of the skin, pupils largely dilated—the symptoms resembling the congestive form of intermitting fever which we frequently meet with in this section of country.

The third modification of the disease, where the irritation is directed to the alimentary canal, was of common occurrence in the epidemic of 1848–9, and was sometimes rapidly fatal. Like the other forms, this may be sudden in its attack. The child is seized with vomiting; diarrhœa soon follows, the discharges being frequent, copious, and watery, at first of a yellow color, soon becoming almost clear. The efforts at vomiting are frequent; the thirst constant; patches of a livid-colored rash can sometimes be indistinctly seen about the neck or over different parts of the body; the tongue is moist and heavily coated; the throat slightly inflamed; the pulse remarkably small, feeble, and frequent; respiration labored and irregular, frequent disposition to make a long inspiration; the countenance contracted, and the eyes sunken. Death sometimes takes place in from ten to twelve hours; the symptoms often very much resembling those of cholera. We had a number of cases of this character following each other in rapid succession. They occurred in January, a season of the year when we could not consider the disease as complicated with cholera infantum, which is seldom prevalent, except in the summer and autumn. A few cases may assist in illustrating this form of the disease.

Columbus Baily, aged six years, was attacked, January 26th, 1849, with vomiting, about five o'clock in the morning; he soon afterwards had diarrhœa. His mother gave him some laudanum and camphor, which appeared to check the disease. During the forenoon he was slightly feverish, and an indistinct appearance of rash could be seen on the forehead, neck, and breast; at half-past twelve o'clock he was again suddenly attacked with vomiting and purging. At one o'clock I was sent for. Shortly after I entered the room he had a profuse serous discharge from his bowels, an unusually large operation for so small a child. The vomiting was very frequent and distressing; the pulse small and rapid; the countenance pale and shrunken; thirst almost incessant; tongue coated and moist; the eyes were sunken; the skin became cold, and the child sunk into collapse, and expired in a slight convulsion about nine o'clock in the evening. His mother informed me that he appeared perfectly well the day before. I treated the case with stimulants, anodynes, astringents, counter-irritants, &c. &c. Had cholera been prevailing in this section of country, I should certainly have thought this case complicated with that disease.

A few days after the occurrence of this case, David C., between four and five years of age, son of the Rev. J. C., of this town, was attacked about four o'clock in the morning with vomiting and purging. When I saw him, about seven o'clock, his pulse was remarkably rapid; vomiting and purging frequent; discharges serous; tongue moist; throat slightly inflamed, of rather a purple color; mind clear. During the day there was an indistinct appearance of rash over different parts of the body, giving the skin a mottled appearance. The symptoms of prostration continued to increase, and the child died about eight o'clock in the evening. This patient appeared well the day before, and ate his evening meal as usual.

On the 23d of January, 1849, Mr. J. Noble, of this town, called on me to attend his daughter, aged eight years. The child was attacked suddenly with vomiting and purging; her pulse was small and feeble; the skin was cold, and had a livid appearance; there was great prostration, restlessness, incessant thirst, and nearly all the symptoms described in the other cases. Mr. N. informed me, that six days previous he lost a daughter five years old, with almost precisely the same symptoms, after about ten hours' illness. I resorted to an active course of treatment with stimulants, astringents, and revulsives, and, contrary to what I expected, reaction took place; the rash made its appearance, though not very generally, over the body, and the patient passed through the disease more favorably than many cases did that commenced with milder symptoms. We had a number of cases of this form of the disease. The rash seldom came out well, and in those that recovered there was but little exfoliation of the cuticle. It appeared to me that in all the forms of malignant scarlatina, the sudden depression of the system generally occurred *just as the rash was making its appearance*, and in this form, where the violence of the disease was directed to the alimentary canal, I could not avoid the conclusion, when I saw sometimes in the same family the rash coming out over the whole mucous membrane of the mouth and fauces, and the epithelium of the tongue peeling off, that the rash might also be directed to the mucous membrane of the alimentary canal and produce a violent form of gastro-enteritis, or a peculiar form of congestive diarrhœa.

Wilson states, in his work on Diseases of the Skin, that "The primary fever of scarlatina maligna is of longer duration than that of the two preceding forms of the disease; the cutaneous eruption not appearing until the third or fourth day." This, I think, is not always the case. I have seen the rash appear on what I thought was the first day of the attack. Wood, in his Practice of Medicine, says that the rash makes its appearance "sometimes at the very commencement, so far as can be determined from statements of the patient or his friends." This agrees with my experience, and I think will apply to all forms of the disease.

The fourth form of malignant scarlatina, where the violence of the malady is directed to the throat, is the usual form of the disease, and is so accurately described in our medical books that it would be useless to give a repetition of the symptoms. It differs as widely in its symptoms from the other modifications as they do from each other.

These appear to be the most distinct or extreme forms of scarlatina maligna. Subdivisions of this disease may probably be considered unnecessary, and perhaps they are, but if these divisions assist in directing attention to the different appearances which this malady occasionally presents, or in any way throw one ray of additional light upon a disease which has destroyed its myriads, and which has not yet ceased from its work of death, the object for which they were made will have been accomplished.

It is scarcely necessary to mention, that these forms may be met with under various combinations, presenting a great diversity of symptoms.

Death may occur in scarlatina, 1st. From shock to the nervous system; sudden depression of the vital forces; 2d. From congestion; engorgement of some of the principal organs; 3d. From inflammation, either sthenic or asthenic.

The treatment of a disease so protean in its character must be almost as various as the forms of the malady are different; and cases occur in which the treatment must differ as widely as the treatment of cholera from that of meningitis, or croup from congestive fever. In treating scarlatina, I have regarded the disease as capable of assuming the mild, the inflammatory, and malignant varieties. This division, like the subdivisions in the malignant form, directs attention to the principal modifications in which the disease may appear. The mild, from the little derangement produced in the system, requires but little treatment. The inflammatory, from the violence of the reaction, being often complicated with inflammation of the throat, the brain, or some other part of the system, requires at first an antiphlogistic treatment. The malignant, with the modifications already described, requires treatment adapted to each, and at the same time remedies to sustain the powers of the system.

During six months of almost daily practice in this disease, I tried a great variety of remedies; some, I thought, produced good effects, others did not meet my expectations. Emetics of antimony or ipecacuanha, mercurial or active cathartics, and all medicines which have a tendency to depress the powers of the system, are justly considered dangerous remedies in scarlatina maligna. I have, however, in the low forms of the disease, seen most excellent effects from the salt and mustard emetic, which, I believe, is not a new remedy, although I cannot find it recommended in any of the works that are in my possession. I was induced to try it in the case of a little child that was suddenly prostrated with the disease. This child had vomited and thrown up undigested food, and suspecting that the stomach might be overloaded, and fearing, from the feeble and rapid pulse, to give either antimony or ipecacuanha, I administered an emetic of salt and mustard; this produced a good effect, reaction took place, and the child finally recovered. I afterwards prescribed the same in a number of instances, and its operation has pleased me so well that I now regard it amongst the best remedies we possess in bringing about reaction in the low forms of this disease. When the system was suddenly prostrated at the commencement of the attack with symptoms similar to syncope, a stimulating course of treatment appeared to be the only one indicated, and, with me, was the most successful. A mustard emetic, followed by the hot bath, or friction with the bare hand, dry salt, or cayenne pepper, or plasters of mustard or cayenne pepper over the stomach, spine, and extremities, and the administration of either carbonate of ammonia, quinine, camphor, capsicum, brandy, or an infusion of serpentaria, as the symptoms indicated, were the remedies principally depended upon. It is unnecessary to give the treatment of the disease in detail, as it presents nothing very unusual or very satisfactory; a mere outline will show the course we generally pursued.

When we succeeded in bringing about reaction, sometimes little had to be done, except occasionally to give the acetate of ammonia, or an infusion of serpentaria, or some mild remedy. In other cases, the powers of the system had to be sustained with active stimulants,—quinine, &c. &c.

When the violence of the disease was directed to the brain, if there were symptoms of inflammation, we depended upon local bleeding, cupping the back of the neck, or behind the ears, the temples, &c. Also applied cold to the head; gave cathartics, diaphoretics, and sometimes stimulating enemas; sinapisms were applied, if the rash was not out well, to the extremities, and over different parts of the body. I cannot say that I ever saw much benefit from bloodletting in any form of scarlatina. When there was evidence of congestion of the brain, with cold skin, small and feeble pulse, deep coma, &c., after other remedies had been tried, I have seen benefit from the following treatment: Hold the child over an empty tub; dash three or four pailfuls of cold water over the head in rapid succession; immediately after apply mustard plasters, as hot as can well be borne by the hand, to the back of the neck, throat, back, breast, and arms; let these remain on for a short time, or until they redden the skin; if reaction does not take place in the course of two or three hours, repeat this treatment. In addition, let the feet be immersed in warm water, and administer stimulating enemas.

When the disease was directed to the alimentary canal, I treated it with stimulants, anodynes, astringents, revulsives, and counter-irritants. The hot mustard bath and mustard emetics are remedies from which, I think, I have seen good effects in this form of the disease.

When the throat was the part principally affected, acidulated and astringent gargles or washes, or an infusion of capsicum and vinegar as a wash; and if ulceration existed, the application of a strong solution of the nitrate of silver, were the local remedies principally depended upon. Carbonate of ammonia, brandy, quinine, serpentaria, were also administered in the low form of the disease. The chlorate of potash, I thought produced, in many instances, good effects. These remedies were prescribed as symptoms required; at the same time, the powers of the system were sustained with a light, nutritious diet. The best external application that I know of is a piece of fat bacon. This domestic remedy is generally used by the people in diseases of the throat, and it is one to whose efficacy in scarlatina I can bear testimony. Liniments, sinapisms, or blisters, are either too transient in their effects, or are too severe. This remedy brings out a fine vesicular eruption, which produces a constant and sufficiently irritating effect. A piece of bacon is shaped so as to fit the throat from ear to ear—I generally sprinkle it thickly with salt—and, when once applied, it does not require changing. I now invariably apply this remedy as soon as I am called to attend a case of scarlatina, and I think it frequently prevents the throat from becoming very much affected. I tried inunction, as recommended by Dr. Scheneman, in several cases of the inflammatory form of the disease, but I cannot say that I saw any beneficial effects from this treatment. The form of scarlatina in which the throat is the principal seat of the disease, is not the most fatal, but frequently very lingering, and the rash continues out much longer than in the other varieties.

A specific disease, like scarlatina, which requires a certain period for it to pass through its evolutions, often depressing, at the very commencement, the powers of the system, at the same time giving rise to local inflammations of an asthenic character, must, from its very nature, be difficult to manage, and will too often baffle the best directed efforts of the physician.

Belladonna was given as a prophylactic; but seeing children attacked with the disease while taking the medicine, I lost all confidence in it, and perhaps discontinued it before I had given the medicine a thorough trial.

